# Assessment of the Relationship Between Neutrophil-Lymphocyte Ratio and Dyspeptic Symptoms in Patients With Peptic Ulcer Diagnosed by Endoscopy and Patients Without Peptic Ulcer

**DOI:** 10.7759/cureus.46820

**Published:** 2023-10-10

**Authors:** Süleyman Coşgun, Zülküf Aras

**Affiliations:** 1 Department of Gastroenterology, Kütahya University of Health Sciences, Kütahya, TUR; 2 Department of Internal Medicine, Kütahya University of Health Sciences, Kütahya, TUR

**Keywords:** helıcobacter pylorı, non-ulcer dyspepsia, peptic ulcer, endoscopy, neutrophil/lymphocyte ratio

## Abstract

Introduction: Neutrophil-lymphocyte ratio (NLR), a simple, inexpensive, and effective biomarker associated with various inflammatory and neoplastic diseases, has become the focus of attention in recent years. Nevertheless, it has not been adequately studied in dyspeptic patients with peptic ulcer (PU) and without PU, i.e., patients with non-ulcer dyspepsia (NUD).

Materials and methods: The population of this retrospective study consisted of patients with dyspeptic complaints who underwent esophagogastroduodenoscopy between April 2023 and June 2023. A total of 180 patients included in the study sample were categorized into two groups based on the endoscopy results: patients without PU or NUD patients (Group 1) and those with PU (Group 2). Age, gender and pre-procedural NLR data, upper gastrointestinal endoscopy results, and the presence of *Helicobacter pylori *(Hp) in endoscopic biopsy information were recorded for all patients.

Results: Of the 180 patients, 140 were diagnosed with NUD (Group 1), and 40 were diagnosed with PU (Group 2). There was a significant difference between Groups 1 and 2 in terms of NLR (Group 1: 2.5±1.8 vs. Group 2: 3.6±3.7, p=0.014). There was also a significant difference in NLR between Group 1 and Group Gastric Ulcer (p=0.030) but not between Group 1 and Group Duodenal Ulcer (p=0.064). Rates of patients with positive Hp test results were 25% and 32.5% in Groups 1 and 2, respectively, indicating a significant difference between the groups (p=0.026).

Conclusion: The NLR measured in patients who presented with dyspeptic complaints was found to be significantly higher in PU patients than in NUD patients. The elevated NLR levels were even more evident in PU patients with GU compared to PU patients with DU.

## Introduction

Dyspepsia is a symptom complex characterized by persistent or recurrent pain or discomfort in the upper abdomen that has been present for at least three months [1]. Dyspepsia, the definition of which has changed in the last 50 years, is a frequently encountered complaint in society and in patients who apply to health institutions [2,3]. Many factors, including gastroesophageal reflux disease, peptic ulcer (PU), and functional dyspepsia, may cause dyspepsia [4,5].

Neutrophil-lymphocyte ratio (NLR), a simple, inexpensive, and effective biomarker associated with various inflammatory and neoplastic diseases, has become the focus of attention in recent years [6,7]. Nevertheless, NLR has not been adequately studied in dyspeptic PU patients and dyspeptic patients without PU, i.e., patients with non-ulcer dyspepsia (NUD). In this context, the objective of this study is to assess the relationship between pre-procedural serum NLR and dyspeptic symptoms in patients with PU diagnosed by esophagogastroduodenoscopy and in NUD patients.

## Materials and methods

The population of this retrospective study consisted of the patients who underwent esophagogastroduodenoscopy at Kütahya Health Sciences University Evliya Çelebi Training and Research Hospital Gastroenterology Clinic due to dyspeptic complaints in 2023. Patients with dyspeptic complaints were selected by taking into account the symptoms used to diagnose dyspepsia in the Rome IV criteria [8]. Accordingly, patients with one or more complaints of postprandial fullness, early satiety, epigastric pain, and epigastric burning were included in the study. Informed consent was obtained from all the participants, and procedures were conducted according to the Declaration of Helsinki. This study protocol was reviewed and approved by the Kütahya Health Science University Clinical Trials Ethical Committee, approval number: 2023/03-06. Patients who had major abdominal surgery, biliary system disease, esophagitis, gastric atrophy with esophageal varices, a history of systemic metabolic disease, and signs of active infection were excluded from the study. In the end, a total of 180 patients were included in the study sample. These patients were categorized into two groups based on the endoscopy results: the patients without PU or NUD patients (Group 1, n=140) and those with PU (Group 2, n=40). The 40 PU patients were further categorized into two subgroups: gastric ulcer (GU Group, n=21) and duodenal ulcer (DU Group, n=19). A single clinician performed esophagogastroduodenoscopies in all patients included in the study. Biopsies were taken from the antrum during the procedure and then sent for histopathological examination to check for the presence of *Helicobacter pylori* (Hp). The patients' hemogram (complete blood count) tests taken before endoscopy were examined and NLR was evaluated.

All statistical analyses were performed using the SPPS 22.0 (Statistical Product and Service Solutions for Windows, Version 22.0, IBM Corp., Armonk, NY) software package. Continuous variables were expressed as mean±standard deviation values, whereas the categorical variables were expressed as percentage (%) values. Student's t-test and Pearson’s chi-squared test were used to compare continuous and categorical variables, respectively. Probability (p) statistics ≤0.05 were deemed to indicate statistical significance.

## Results

Of the 180 patients included in the study, 140 had NUD (Group 1), and 40 had PU (Group 2). Of the 40 PU patients, 21 had gastric ulcers (GU Group) and 19 had duodenal ulcers (DU Group). While no significant difference was found between Groups 1 and 2 in mean age, there was a significant difference in gender distribution (p=0.000). NUD was more common in women and PU in men. The distribution of the demographic characteristics of the patients by the groups is shown in Table 1.

**Table 1:** Clinical and demographic characteristics of both groups

	Groups		
	Group 1 (n=140)	Group 2 (n=40)	p-value
Age	53.3 ± 15.3	57.0 ± 17.0	0.196
Gender			
Male (n=84)	54	30	<0.001
Female (n=96)	86	10	

The comparison of NLR between Groups 1 and 2 revealed that NLR values were significantly more elevated in Group 2 than in Group 1 (Group 1: 2.5±1.8 vs. Group 2: 3.6±3.7, p=0.014). There was also a significant difference in NLR between Group 1 and Group GU (p=0.030) but not between Group 1 and Group DU (p=0.064). There was no significant difference between Group GU and Group DU in NLR. The distribution of NLR by the groups is given in Table 2.

**Table 2:** Comparison of NUD patients with Group 2 (PU) and other subgroups, Neutrophil-lymphocyte ratio NUD: non-ulcer dyspepsia, PU: peptic ulcer

	Non-ulcer dyspepsia (n=140)	Peptic ulcer (n=40)	Gastric ulcer (n=21)	Duodenal ulcer (n=19)
Neutrophil-to-lymphocyte ratio	2.5 ± 1.8	3.6 ± 3.7	3.7 ± 4.4	3.5 ± 2.9

In the histopathological examination, the rate of Hp-positive patients was 25% and 32.5% in Group 1 and Group 2, respectively, and a significant difference was found between the groups (p = 0.026). In addition, while Hp positivity was 48% in patients with stomach ulcers (GU Group), it was 66% in patients with duodenal ulcers (DU Group), and a significant difference was found (p = 0.00.4). However, there was no significant difference in NLR between those with and without a positive Hp test result (mean NLR of Hp-positive patients: 2.4±1.0 vs. mean NLR of Hp-negative patients: 2.7±2.2, p=0.324).

## Discussion

A problem that is encountered every day in gastroenterology practice and needs to be addressed is whether a patient who presented with unexplored dyspepsia has an ulcer. The fact that endoscopy, an invasive procedure, is required to diagnose ulcers is usually not welcomed by patients.

Due to the presence of organic causes in approximately 40% of dyspeptic cases, some researchers argue that endoscopy and abdominal ultrasonography should be performed on all patients presenting with dyspeptic complaints before they are started on any empirical treatment [9]. In parallel, it was suggested in some cost analysis studies that performing endoscopy before treatment, in fact, reduces the overall costs [10]. On the other hand, given that as high as 25% or more of the general population has dyspeptic symptoms and that endoscopy is an expensive technique, it was stated in other studies that many factors should be evaluated before making the decision to perform endoscopy prior to starting patients on any treatment [11].

In this context, this study was designed with the hypothesis that NLR, a biochemically measurable biomarker, may help identify the patients who require endoscopy more than others by predicting the presence of ulcers and the patients who should be more actively recommended to undergo an endoscopy after the tests-therapeutic treatments were not successful.

Given that it is a simple, inexpensive, and effective biomarker associated with various inflammatory and neoplastic diseases, NLR has become popular in recent years as an indicator of subclinical inflammation [12,13].

Along these lines, the patients who presented to the clinic with unexplored dyspepsia and had biochemical tests and esophagogastroduodenoscopy were retrospectively reviewed in this study, and the endoscopic findings, biopsy results, and the Hp test results thereof were compared to pre-procedural serum NLR values.

The results indicated a significant difference between Groups 1 and 2 in pre-procedural serum NLR values. This difference can be attributed to the fact that the ulcers in PU patients developed on top of gastritis and featured a more inflammatory process arising from both gastritis and ulcers [14].

The PU patients, especially those with GU, had significantly higher NLR values. In fact, the mean NLR value of even the patients who do not have ulcers, i.e., NUD patients, was 2.5, a quite high value. This result can be explained by the fact that these patients had gastritis with dyspeptic symptoms, even though they do not have ulcers. As a matter of fact, Farah et al. [15] reported quite low NLR levels compared to the levels found in this study in a study involving asymptomatic and symptomatic cases.

Farah et al. demonstrated a significant difference in NLR values between 50 Hp-positive dyspeptic cases and 50 Hp-negative healthy cases. In comparison, in this study, there was no significant difference in NLR values between the dyspeptic patients with and without a positive Hp test result. This result can be attributed to the presence of gastric inflammation in dyspepsia patients, regardless of the presence of Hp in the etiology.

In addition, the inflammation was higher in the GU group than in the DU group, which was expected.

In sum, the study findings suggest that NLR has a predictive value in the differentiation of NUD and ulcers in patients with dyspepsia.

The most important limiting factors of our study are that it was single-center and was conducted with a relatively small number of patients.

## Conclusions

In conclusion, the NLR levels measured before endoscopy were significantly higher in PU patients than in NUD patients with dyspepsia. However, given the study's retrospective design, its findings could not be evaluated in comparison with the results that would otherwise be obtained from an asymptomatic control group. For this reason, further large-scale studies involving an asymptomatic control group are needed to shed more light on the relationship between organic/functional dyspepsia and NLR.
